# Optical trapping reveals propulsion forces, power generation and motility efficiency of the unicellular parasites *Trypanosoma brucei brucei*

**DOI:** 10.1038/srep06515

**Published:** 2014-10-01

**Authors:** Eric Stellamanns, Sravanti Uppaluri, Axel Hochstetter, Niko Heddergott, Markus Engstler, Thomas Pfohl

**Affiliations:** 1 Department of Complex Fluids, Max Planck Institute for Dynamics and Self-Organization, 37073 Göttingen, Germany; 2 Department of Chemistry, University of Basel, 4056 Basel, Switzerland; 3 Department of Cell and Developmental Biology, Biocentre, University of Würzburg, 97074 Würzburg, Germany; 4 Current address: DESY Photon Science, 22607 Hamburg, Germany.; 5 Current address: Department of Chemical and Biological Engineering, Princeton University, Princeton, NJ 08544, USA.

## Abstract

Unicellular parasites have developed sophisticated swimming mechanisms to survive in a wide range of environments. Cell motility of African trypanosomes, parasites responsible for fatal illness in humans and animals, is crucial both in the insect vector and the mammalian host. Using millisecond-scale imaging in a microfluidics platform along with a custom made optical trap, we are able to confine single cells to study trypanosome motility. From the trapping characteristics of the cells, we determine the propulsion force generated by cells with a single flagellum as well as of dividing trypanosomes with two fully developed flagella. Estimates of the dissipative energy and the power generation of single cells obtained from the motility patterns of the trypanosomes within the optical trap indicate that specific motility characteristics, in addition to locomotion, may be required for antibody clearance. Introducing a steerable second optical trap we could further measure the force, which is generated at the flagellar tip. Differences in the cellular structure of the trypanosomes are correlated with the trapping and motility characteristics and in consequence with their propulsion force, dissipative energy and power generation.

Eukaryotes have evolved several mechanisms for locomotion, which critically affect the dynamics of cell division, proliferation, development, and viability^1^,^2^,^3^,^4^. Beyond an understanding of the physiological functions of cellular motion, single cell motility studies reveal biophysical insights to life at the micro-scale, dominated by friction forces^5^,^6^,^7^,^8^. In this context, *Trypanosoma* are a unique model system for motility studies^9^. Trypanosomes are unicellular parasites that infect mammalian hosts, potentially causing death in endemic areas lacking medical treatment options. Introduced into the bloodstream through the bite of a tsetse fly, African trypanosomes propel themselves utilizing a flagellum that extends along the entire cell body from the flagellar pocket to the anterior end of the cell where it rotates freely (see figure 1 for illustration). Hydrodynamic drag during propulsion allows trypanosomes to internalize surface-bound antibodies, thus mediating escape from immune attack by the host^10^,^11^. Prior studies have characterized trypanosome propulsion as a motion similar to a corkscrew, where the flagellar pulse is transmitted from the tip of the flagellum to the base of the cell body^12^,^13^,^14^. Recently it has been shown that trypanosomes are propelled forward by a quasi-planar beat of the flagellum and that maximum forward velocity can be achieved in the presence of microscale particles and obstacles such as in blood flow. At high densities of obstacles with tight interspaces, a reversal of the flagellar beat occurs and the cells swim backwards in order to avoid trapping^15^. The bloodstream form (BSF) of trypanosomes proliferate within the bloodstream, travel along shear gradients, penetrate tissues, and ultimately bypass the blood brain barrier, invading the central nervous system^15^,^16^,^17^. Despite the importance of propulsion in the trypanosome's life cycle, a conclusive physical description of their motility and the molecular players involved is lacking.

Optical traps and tweezers provide an effective means for the manipulation of atoms and mesoscopic objects, including beads and organelles, as well as, biological cells and objects with lengths exceeding 0.1 mm^18^. We integrate a microfluidics platform and an optical trap with high-resolution imaging to investigate the nanomechanics and motility of trypanosomes, yielding physical parameters that could be related to the transmission and infection statistics^19^,^20^,^21^,^22^.

By employing a near infrared optical point trap, living trypanosomes can be captured at a fixed position and manipulated for several minutes. The trapping strengths and locations are correlated with structural and morphological properties of trypanosome cells. Observing the mobility patterns of optically confined cells allows us to quantify the propulsion force, the generated power, and the dissipated metabolic energy at the single cell level.

## Results

### Optical trapping of trypanosomes

**Figure 1:**
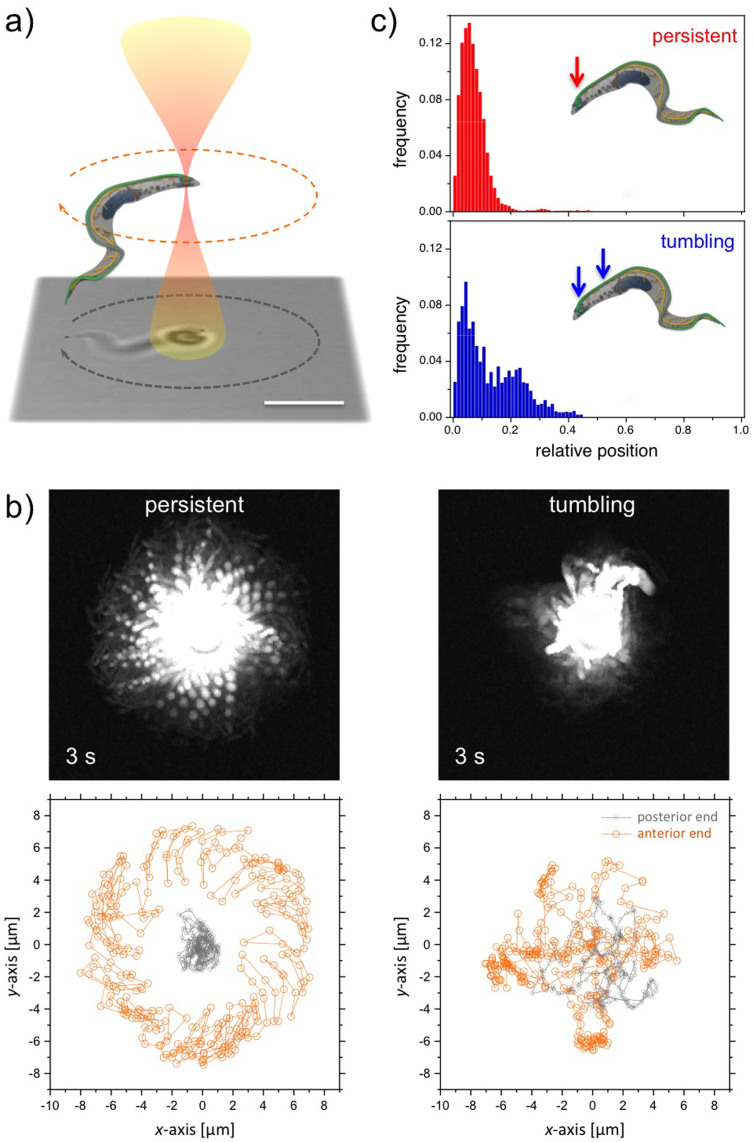
Optical trapping of trypanosomes: (a) Schematic representation of an optical tweezers setup for trapping motile trypanosomes. Within the optical trap the motility is limited, whereas the mobility is unaffected. (b) Overlay of images (3 s, frame rate 100 Hz) from persistent and tumbling walkers in the optical trap. Trajectories of the posterior and anterior end of persistent and tumbling trypanosomes are displayed in the lower part. (c) Histograms of tapping loci of persistent walker and tumbling cells versus the position from the posterior end relative to their contour length *L*. The main trapping positions indicated by arrows are at the posterior end close to the flagellar pocket.

Optical traps have been used widely for the manipulation of atoms, molecules, colloidal particles, organelles, bacteria, and eukaryotic cells^20^. We used a single optical trap at a wavelength in the near IR of *λ* = 808 nm to spatially confine and manipulate the unicellular parasite *Trypanosoma brucei brucei* in its wild-type bloodstream form (BSF). Trypanosomes are highly motile cells with a rapidly deforming asymmetric body with a length of *L* ≈ 20 μm and a width of *d* ≈ 3 μm. The strong shape deformations are caused by the beating flagellum, which is attached to the length of the cell body. Despite the size and asymmetry of the cell body, we observe that motile trypanosomes can be effectively manipulated and spatially confined using a strongly focused laser beam. Motile trypanosomes that are dragged into the optical trap, maintain mobility with strong shape deformations, since the focal volume of an optical trap (≈0.04 μm^3^) is very small in comparison to the volume of a trypanosome (≈100 μm^3^). Whilst the lateral displacements of trapped trypanosomes are limited, the strong shape deformations are largely unaffected in a focused single beam optical trap (figure 1a). It is possible to optically confine trypanosomes, using relatively weak laser powers of ~10 mW, but even at the highest used laser power of 27 mW, we observe continued cell viability even for trapping periods exceeding 60 min. Therefore, phototoxic effects are negligible on timescales < 15 min. These observation times are two orders of magnitude greater than the longest correlation times in trypanosome motility^23^. The sample heating in our experimental optical trap setup is smaller than 0.1 K and can be considered negligible^24^.

Trypanosomes that are held in the optical trap perform one of two motions around the centre of the trap: 1) an irregular, or 2) a clockwise rotating motion (figure 1b). Trypanosomes that rotate in the optical trap and have periodic motility patterns always show a highly directional motion like a “persistent walker” under non-trapping conditions when they are released from the trap^23^. On the other hand, trypanosomes, which randomly writhe within the optical trap, can be characterized outside the optical trap as “tumbling walkers” with no persistence in direction and a random occurrence of flagellar beat reversal^15^,^23^.

In figure 1c, histograms of trapping loci of tumbling and persistent walker cells versus the distance to the posterior end relative to their contour length *L* are presented. The trapping positions of persistent walkers are more localized than of tumbling walkers. A sliding between the two preferred loci is observed for tumbling trypanosomes. Nevertheless, both cell types have almost the same main trapping area at the posterior end, which indicates a specific structure within the cells that is favourable for optical trapping. The main trapping locus coincides with the kinetoplast near the flagellar pocket, located in the posterior part of the cell (figure 1c). This is consistent with the relatively high refractive index of DNA – the primary component of the kinetoplast, which harbours the mitochondrial genome of the parasite.

### Propulsion force of trypanosomes

**Figure 2:**
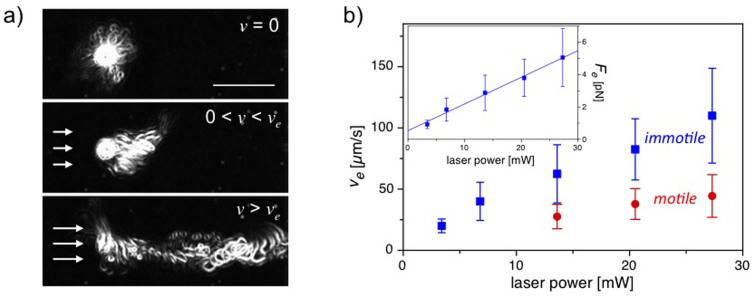
(a) Overlay of images of trapped trypanosomes in different flow conditions. The trypanosomes are dragged out of the optical trap at flow velocities of *v_e_*. Bar represents 20 μm. (b) Plot of *v_e_* versus the applied laser power for motile and immotile cells. Calibration curve of the stall forces *F_e_* versus the applied laser power are shown in the inset.

Living trypanosomes were successfully trapped within a microfluidic flow channel at different laser powers. A minimum laser power of 13 mW was needed to continuously trap a motile trypanosome. Linear flow ramps were repeatedly applied and the cells were recorded until they got dragged out of the trap (figure 2). The resulting cell traces were used to determine the actual flow velocity *v_e_* at which the cells got dragged out of the trap. Escape velocities *v_e_* for different laser powers are shown in figure 2b. Observing the cell behaviour within the optical trap, we found a flow-induced alignment with increasing flow velocities *v* (figure 2a). For a laser power of about 27 mW at the sample, trypanosomes are able to rotate freely within the boundaries of the optical confinement without applied flow (*v* = 0 μm/s). With an applied flow, the cells align parallel to the flow direction. With increasing the flow velocity, the cells are dragged out of the trap at an escape velocity *v_e_* (e.g. ≈40 μm/s at a laser power of 27 mW). Both cell orientations, anterior or posterior end first, have been observed, but the major axis is always directed parallel to the flow. In a previous publication, we have shown that non-trapped trypanosomes tend to align parallel to the flow with the flagellar tip pointing upstream at moderate flow velocities of <0.5 mm/s^25^.

Owing to the self-propulsion and active force generation of motile trypanosomes, an analysis of the escape flow velocity *v_e_* depending on the laser power cannot be directly used to determine the stall force of the optical trap by calculating the drag force. Therefore, the stall force of our optical trap was calibrated by using paralyzed trypanosomes. Cell paralysis with 2-deoxy-*D*-glucose as a glucose substitute is reversible for incubation times less than 15 min^25^,^26^, while the overall cell structure and shape remain unchanged. Consequently, the applied optical forces should be comparable to experiments with motile cells and can be taken as a reference for motile cells. Escape flow velocities *v_e_* for paralyzed trypanosomes for different laser powers are shown in figure 2b. In comparison to *v_e_* for paralyzed cells, the escape flow velocities of motile trypanosomes are smaller owing to the additional propulsion of the motile cells. The paralyzed cells are also aligned with their major axis directed parallel to the flow, before they get dragged out of the optical trap (supplementary information S1). With escape flow velocities *v_e_* and the friction coefficients *ξ* of the cells, we are able to calculate the stall forces *F_e_* = *v_e_ξ*. The parallel translational friction coefficient *ξ_tp_* of a rod with the dimensions of escaping cells is used to approximate the anisotropic friction coefficient of trypanosomes, 
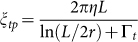
, with length *L* of the rod, diameter 2*r* of the rod, the end correction factor Γ*_t_* (≈0.25 for the measured *L*/2*r* ratio) and the viscosity of the buffer solution *η*^27^. The obtained stall forces *F_e_* using a friction coefficient of *ξ_tp_* = 4.6·10^−8^ kg/s versus the laser power is plotted in the inset of figure 2b. The slope of the linear fit allows us to measure the stall force of the optical trap at (0.165 ± 0.009) pN/mW on the trypanosome.

**Figure 3:**
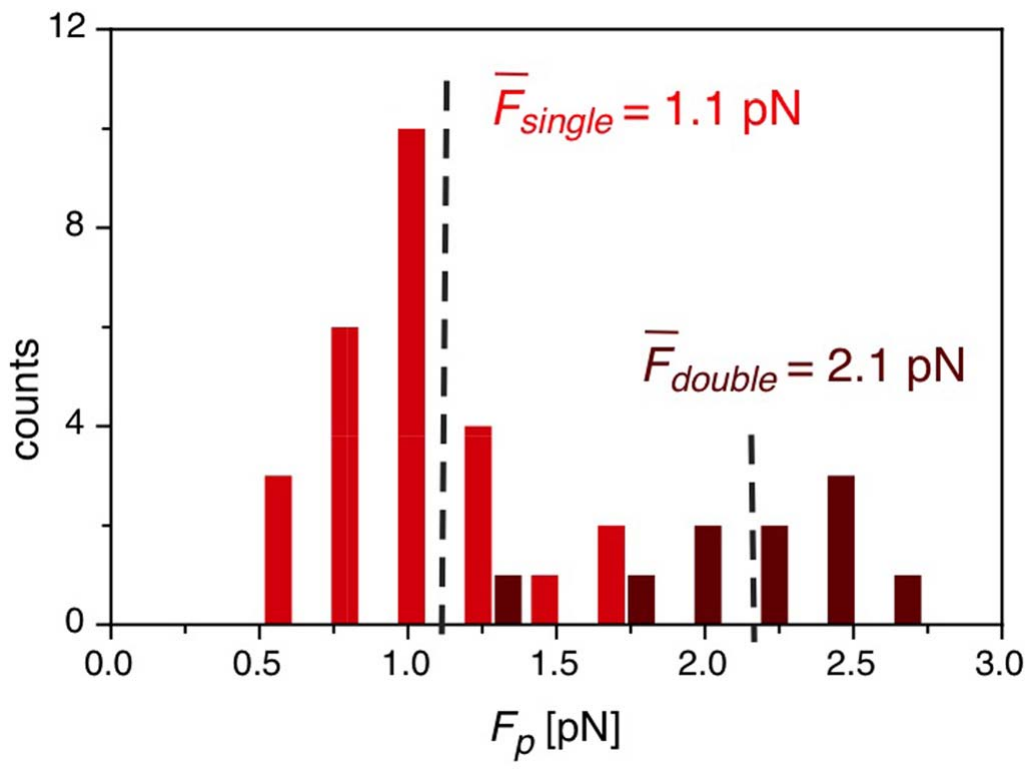
Histograms of measured propulsion forces *F_p_* of trypanosomes with one and with two fully developed flagella.

In order to measure the propulsion force of motile trypanosomes, the cells are initially trapped under no flow conditions (*v* = 0 μm/s) with a laser power of ~20 mW. After cell trapping, the laser power is subsequently reduced by 1 mW steps every 5 s and the applied laser power is recorded at the moment of cell escape from the optical trap. The measured propulsion forces of motile trypanosomes are presented in figure 3. In addition to cells with a single flagellum, cells with two visible beating flagella are analysed as well. The process of fission-based duplication of proliferating trypanosomes is accompanied by the growth of a second flagellum. At the end of the growth phase of the second flagellum shortly before cell division, the trypanosomes exist as doublets^28^. The histograms of propulsion forces show clearly different distributions for cells with a single flagellum and cells with fully developed two flagella. We measure an average propulsion force of 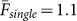 for motile cells with a single flagellum and a nearly two-fold increase in propulsion force of 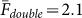 for cells with two flagella. The forces in each of the flagella in the doublet appear to be nearly equal and are additive in order to escape the optical trap. This behaviour may play an important role in the final cleaving stage during cell fission following replication, propagation and ultimately tissue invasion.

### Spectral power of trapped trypanosomes

**Figure 4:**
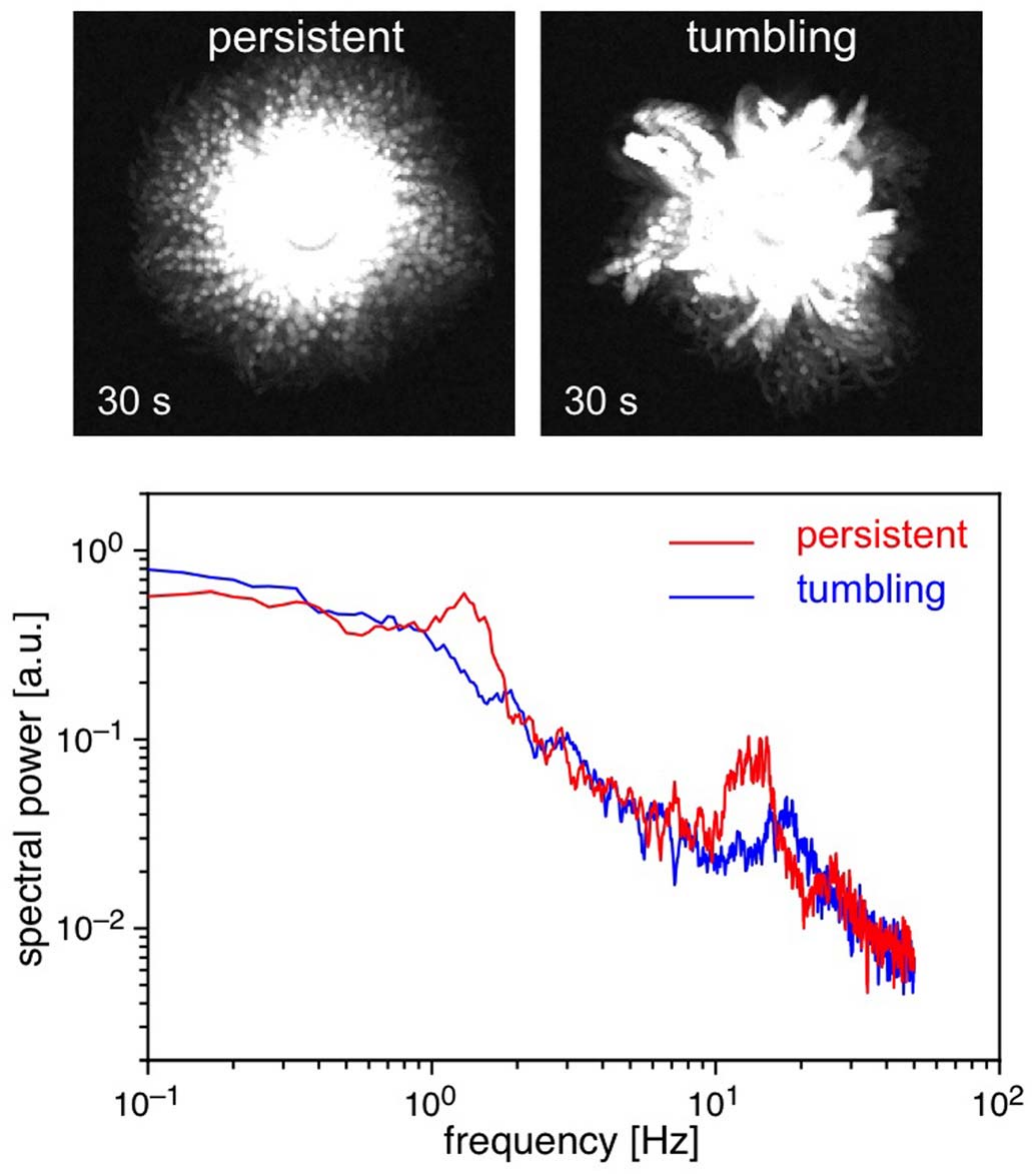
Overlay of images (>30 s, frame rate 100 Hz) from persistent and tumbling walkers. The power spectra of the centre of mass displacement have three peaks in case of rotating, persistent cells and only one peak in case of tumbling cells.

The temporal patterns of trypanosome motility can be characterized by the time-dependent analysis of the centre of mass displacement. Typical power spectra, which are obtained by determining the Fourier transform of the temporal autocorrelation functions of the centre of mass displacement, are presented in figure 4. The power spectrum of a trapped rotating, persistent walker trypanosome reveals three characteristic peaks with frequencies of *f*_Ω_ ~ 1 Hz, *f_ω_* ~ 15 Hz and *f*_2*ω*_ ~ 30 Hz. The first frequency *f*_Ω_ corresponds to the rotation of trypanosomes in the trap, *f_ω_* is the frequency of a flagellar beat and *f*_2*ω*_ is twice the flagellar beat frequency. Since there is no dominant rotation frequency, the power spectrum of a trapped tumbling trypanosome shows only a single frequency *f_ω_* ~ 15–20 Hz corresponding to the frequency of a flagellar beat. The measured flagella frequencies *f_ω_* for rotating and tumbling trypanosomes are consistent with those observed in experiments with freely swimming cells^15^,^23^,^29^, demonstrating that the optical trap did not inhibit or interfere with the cell's mobility.

### Dissipative energy of trypanosomes

Whereas the propulsion of random walker cells induces only an irregular motion in the optical trap, the persistent walker cells perform a continuous rotation. Owing to the optical confinement, the motility patterns of the swimming trypanosomes are translated into a continuous rotation of the cells with a beating anterior flagellum tip. This behaviour is consistent with a quasi-planar beat of the single flagellum described by a straightforward resistive force theory^6^,^15^ and is not in line with a bihelical propulsion mechanism^29^. The movements of the flagellum tip have a mean amplitude of ~4–5 μm and a frequency of about 15 beats per rotation.

Since the energy in our system is dissipated by friction with the surrounded solution, the flagellar beats and rotations allow us to determine the work and power generated by swimming persistent cells.

**Figure 5:**
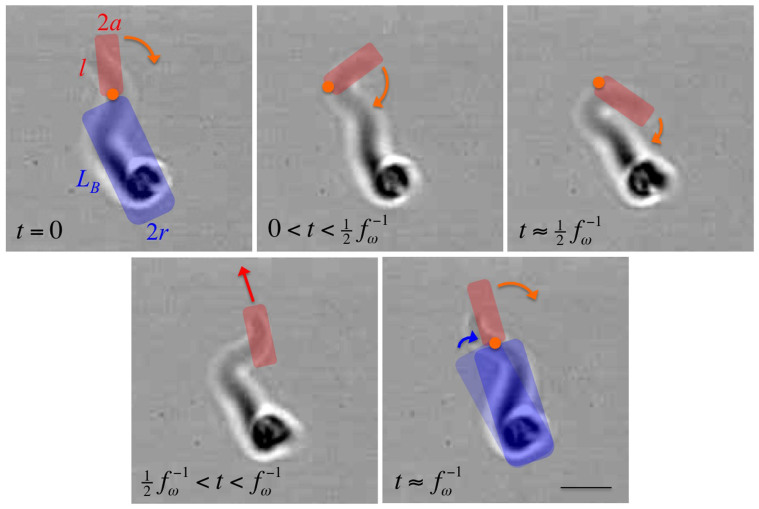
Picture sequence of a flagellar beat of a trapped trypanosome. A sketch of the used rotating and beating rod model is overlaid. Bar represents 5 μm.

For a straightforward estimate of the dissipated energy, we approximate the rotation patterns of the trypanosomes by a rotation of the cell body described as a rod and a beating tip described by a small rod with a combined rotational and translational motion (figure 5). The work for one full body rotation of 2*π* is *W_B_* = 4*π*^2^*ξ_rB_f*_Ω_ and the generated power is 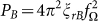. The friction coefficient of the rotating body described by a rod of length *L_B_* and diameter 2*r* is 
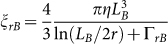
 with Γ*_rB_* the end correction factor^30^,^31^. The motion of the flagellum tip during a single beat is approximated by a rotation of the tip of about *π*, described by a small rod of length *l* and diameter 2*a*, and a subsequent movement of about one tip length *l* in translational direction. In agreement with the experiments, the time intervals of the rotation and translation steps are almost equal with 
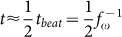
. The energy of the flagellar beat is the sum of the energy for the rotation of ~*π* and the translation of the tip by *l* with a velocity *v_t_* = *l*·2*f_ω_*: 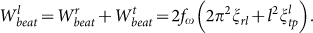


The friction coefficient for the rotating small rod is 
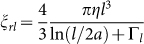
 and the parallel translational friction coefficient is 
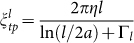
, with the end correction factor Γ*_l_*. For the generated power of the flagellum tip we obtain 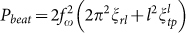. The generated power of rotating persistent runner cells is the sum of both fractions, the flagellar tip and the cell body, *P* = *P_beat_* + *P_B_*. Since 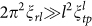, we find following simplified relation, 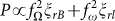. Using the measured experimental values, we calculate *ξ_rB_* = 2.0·10^−18^ kg·m^2^/s, *ξ_rl_* = 6.2·10^−20^ kg·m^2^/s and 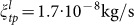. For a typical persistent walking cell, we obtain *W_B_* = 7.9·10^−17^ J for one rotation of the body and 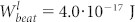 for one stroke of the flagellum tip. The power generated by a trypanosome is about *P* = 6.7·10^−16^ W, with the fractions *P_B_* = 7.9·10^−17^ W for the rotating cell body and of *P_beat_* = 5.9·10^−16^ W for the beating tip. The rate *P_B_*/*P* may be used as a measure of the motility efficiency, comparing the necessary power to rotate the body versus the actual generated power. We observe a *P_B_*/*P* ~ 0.1–0.15, which means that about 7–10 times more power is generated as it is necessary to move a trypanosome. This relatively low power efficiency might be an indication that the motility mechanism is not only designed for locomotion or self propulsion of the trypanosomes but rather that motility is also essential for the removal of antibodies to escape from immune attack by the host as shown by Engstler *et al.*^10^.

### Trypanosomal bead displacement

**Figure 6:**
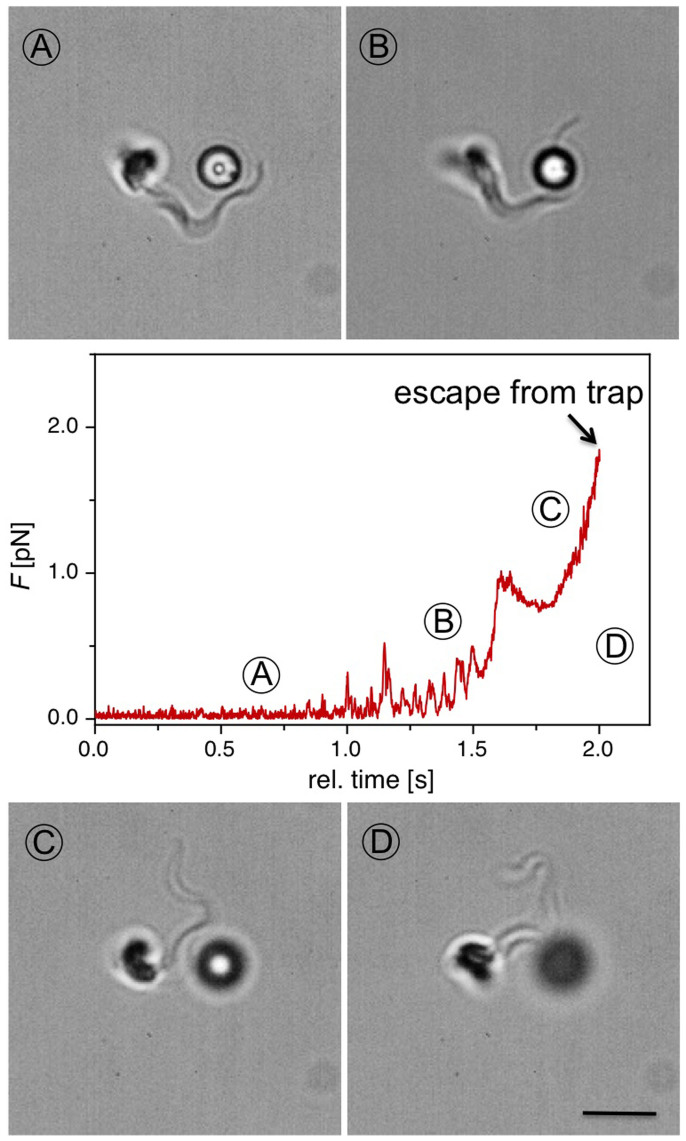
Picture sequence of a trapped trypanosome hitting a colloidal particle caught by a second optical trap (distance between the two traps: 4 μm). The applied force on the bead is calculated by analysing the bead displacement in the second optical trap. Bar represents 4 μm.

To illustrate the potential of optical trapping methods for motility analysis of unicellular swimmers, we modified our experiments and added a second optical trap (*λ* = 808 nm) in our setup, which can be independently controlled. With the first optical trap a motile trypanosome is captured, and a polystyrene bead of radius *d* = 1.5 μm is confined in the second trap. The two traps can be brought in close proximity with lateral distances between the two trapping centres ranging from 1 to 25 μm. A sequence of a rotating trypanosome hitting a polystyrene bead, which is positioned 4 μm from the trapping centre of a trypanosome, is shown in figure 6. The anterior end of the flagellum passes by and touches the bead, which is displaced slightly. After a few stronger hits from the flagellum, the bead is pushed out of the optical trap. The evolution of the applied force on the bead, which can be calculated by analysing the bead displacement in *x* and *y*, and by analysing the intensity change of the first order diffraction peak in *z*-direction for the second trap, is plotted in the graph of figure 6. After a few weak hits of the flagellum with measured forces less than 1 pN, the polystyrene bead is kicked out of the optical potential with a measured force >3.75 pN, the maximum stall force, and the particle diffuses away. Thus with this setup, we can directly measure the force generated at the tip of the flagellum. Our measurement of the force exerted at the flagellar tip is nearly two orders of magnitude lower than estimates generated by analysing the deformation of red blood cells by trypanosome flagellum^29^.

## Conclusions

Using an *in situ* optical trapping and live imaging setup, we are able to directly trap trypanosomes and analyse the motility of the cells while being spatially confined. The observed trapping positions and motility of the unicellular parasites are sensitive to the internal cellular structure. Comparing the trap escape behaviour of self-propelling cells with paralyzed cells, we are able to measure the propagation force of single trypanosomes in the pN range. Analysing the propulsion of trypanosomes doublets, which consist of two fully developed flagella, we find an additivity of the generated forces. Furthermore, we are able to directly measure the generated force at the flagellar tip in the <10 pN range using a double optical trap setup. A detailed analysis of the motility patterns of optically trapped cells allows for the determination of the dissipated energy, power generation and propulsion efficiency of single trypanosomes and suggests that the motility mechanism is not only required for locomotion but could also be essential for survival within the host.

The described optical method to study motility patterns, force generation and dissipated energy of trypanosomes may be used to gain further a deeper understanding of cellular development during different cell cycle stages as well as of the impact of drugs and environmental clues, such as extracellular matrix or blood brain barrier mimics^17^, on the cell's metabolism and motility characteristics. Moreover, this experimental methodology may be transferred to analyse different species of trypanosomes, e.g. *Trypanosoma cruzi*, and other unicellular parasites or motile cells.

## Materials

### Optical trapping and imaging setup

For the optical trapping and imaging experiments, we used a custom-built microscope. Two near infrared laser diodes (*λ* = 808 nm, *P* = 200 mW, BlueSky Research) with pre-aligned diffractive optics, correcting for ellipticity and emitting a diffraction limited single mode constant wave laser beam were used as sources for the optical traps. The two lasers were combined by a polarizing beam splitter in order to have two independent optical traps for simultaneous manipulation of multiple cells or objects. The lasers were integrated into the optical path of the microscope and into the objective, Zeiss EpiPlan Neofluar 100×/oil N.A. 1.3, by a dichroic mirror reflective for the infrared but transmittive for both excitation and emission wavelengths of common fluorescence and bright-field microscopy. The imaging part consisted of a high-speed Vision Research Miro III camera, capable of recording with 120 kHz at 32 × 32 pixels and with 1.2 kHz at full frame mode (800 × 600 pixels). A high power single chip LED (Cree) was integrated for sufficient illumination. The stiffness of the optical traps were calibrated with polystyrene beads of defined diameters by the Stokes drag method or by recording the displacement power spectra at frame rates of about 20 kHz for all three axes.

All optical experiments on trypanosomes have been conducted within PDMS-based microfluidics flow channels of a width of 500 μm and a height of 65 μm. The cells have been trapped 15–25 μm below the cover slip surface to assure all degrees of free cell motility throughout the entire experiment. For the stall force experiments, linear flow ramps were repeatedly applied via a syringe pump system (Nexus nemsys) and the cells were recorded until they got dragged out of the trap. The recorded images were contrast enhanced and background corrected. Custom-made ImageJ (NIH, Bethesda, USA) macros were utilized to determine the center of mass of the trypanosomes and to trace the cell's posterior and anterior ends.

### Preparation of the cells

*Trypanosoma brucei brucei*, strain Lister 427, clone 221a (MITat 1.2), were cultivated in HMI-9 medium at 37°C, 5% CO_2_ and harvested at a density of 7·10^5^ cells per mL. Cells were washed thrice in 1 mL TDB and resuspended in 1 mL TDB. The PDMS microfluidics devices were rinsed with 5% w/v BSA in TDB to prevent the cells from sticking to the walls. The experimental time was restricted to 30 min *ex vitro* in order to prevent major metabolic and morphologic changes of the cells.

To immobilize the cells, *Trypanosoma brucei brucei*, strain Lister 427, clone 221a (MITat 1.2), were cultivated in HMI-9 medium at 37°C, 5% CO_2_ and harvested at a density of 7·10^5^ cells per mL. Afterwards the cells were washed thrice in 1 mL TDB and resuspended in 1 ml 2-deoxy-D-glucose TDB and incubated for 15 min^26^. The cells were only analyzed when active motility ceased to appear.

## Author Contributions

E.S., M.E. and T.P. designed the experiments; E.S. and A.H. performed the experiments; S.U., N.H. and M.E. contributed reagents/materials/analysis tools; E.S., A.H. and T.P. analysed data, and E.S., S.U. and T.P. wrote the manuscript.

## Supplementary Material

Supplementary Information Supplementary information
