# Supplementary material for: Optical trapping reveals propulsion forces, power generation and motility efficiency of the unicellular parasites Trypanosoma brucei brucei
Source: Sci Rep. 2014 Oct 1;4:6515. doi: 10.1038/srep06515 (PMC4180810; doi:10.1038/srep06515)
Supplement: Supplementary Information [file srep06515-s1.pdf]

Optical trapping reveals propulsion forces, power generation and motility efficiency of the unicellular parasites *Trypanosoma brucei brucei*

Eric Stellamanns[a,1], Sravanti Uppaluri[a,2], Axel Hochstetter[b], Niko Heddergott[c], Markus Engstler[c], Thomas Pfohl[a,b,3]

[a]Department of Complex Fluids, Max Planck Institute for Dynamics and Self-Organization, 37073 Göttingen, Germany

[b]Department of Chemistry, University of Basel, 4056 Basel, Switzerland

[c]Department of Cell and Developmental Biology, Biocentre, University of Würzburg, 97074 Würzburg, Germany

[1]present address: DESY Photon Science, 22607 Hamburg, Germany

[2]present address: Department of Chemical and Biological Engineering, Princeton University, Princeton, NJ 08544, USA

[3]To whom correspondence may be addressed. E-mail: thomas.pfohl@unibas.ch

Supplementary information

Figure S1: Optical trapping and determining of escape flow velocity v_e for paralyzed trypanosomes

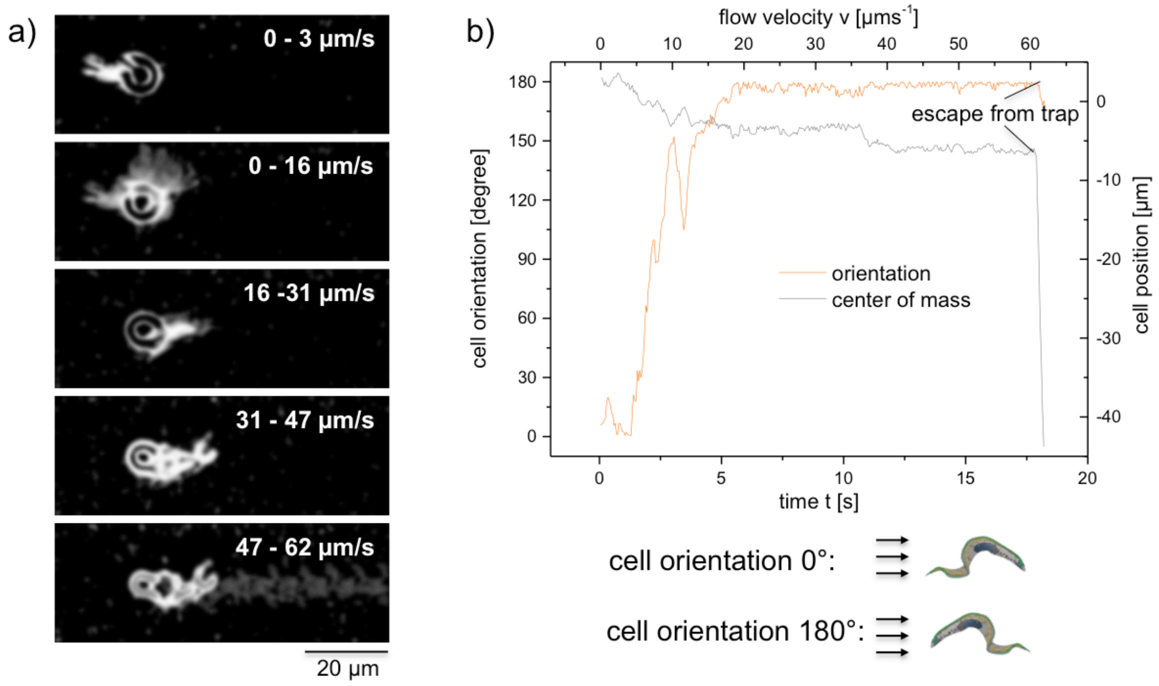

<u>Figure S1:</u> Optical trapping and determining of escape flow velocity v_e for paralyzed trypanosomes

a) Overlay of exemplary images of trapped a paralyzed trypanosome in different flow conditions. The paralyzed trypanosome is dragged out of the optical trap at flow velocities of v_e.

b) Plot of cell orientation and cell position (distance from trap centre) versus flow velocity v (time of flow velocity ramp). The schematic defines orientation is 0° when the cell is facing downstream and 180° when upstream. The escape flow velocity is recorded as the point at which the centre of mass jumps.
